# A Prognostic Model Using Inflammation- and Nutrition-Based Scores in Patients With Metastatic Gastric Adenocarcinoma Treated With Chemotherapy

**DOI:** 10.1097/MD.0000000000003504

**Published:** 2016-04-29

**Authors:** Meng-Che Hsieh, Shih-Hor Wang, Seng-Kee Chuah, Yu-Hung Lin, Jui Lan, Kun-Ming Rau

**Affiliations:** From the Department of Internal Medicine, Division of Hematology-Oncology (M-CH); Department of Surgery, Division of General surgery (S-HW, Y-HL); Department of Internal Medicine, Division of Hepatogastroenterology (S-KC); Department of Pathology (JL), Kaohsiung Chang Gung Memorial Hospital and Chang Gung University College of Medicine; and Gastric cancer team in Kaohsiung Chang Gung Memorial Hospital, Kaohsiung City (M-CH, S-HW, S-KC, Y-HL, JL, K-MR), Taiwan.

## Abstract

The outcomes of patients with metastatic gastric cancer (mGC) are poor. Recent studies have identified the prognostic impact of inflammatory response and nutritional status on survival for patients with gastric cancer. This study aims to create a prognostic model using inflammatory- and nutrition-based scores to predict survival in patients with mGC treated with chemotherapy.

After institutional review board approval, patients who had mGC and were treated with chemotherapy from 2007 to 2012 at Kaohsiung Chang Gung Memorial Hospital were retrospectively reviewed. Significantly predictive factors were identified by multivariate Cox regression analyses. Based on these variables, a prognostic model using inflammatory- and nutrition-based scores was constructed to predict survival. Kaplan-Meier curves were plotted to estimate overall survival. The c-statistic values with 95% confidence interval (CI) were also calculated to access their predicting performances.

Our study consisted of 256 patients with a median age of 60 years and a median follow-up visit of 18.5 months. Multivariate analyses showed that neutrophil to lymphocyte ratio (NLR), modified Glasgow prognostic score (mGPS), and Patient-Generated Subjective Global Assessment (PG-SGA) were independently related to survival. After computing these scores, patients were classified into favorable-, intermediate-, and poor-risk groups. The median overall survival were 27.6 versus 13.2 versus 8.2 months in favorable, intermediate, and poor-risk groups, respectively. The 2-year survival rate was 52% versus 16% versus 3% in favorable-, intermediate-, and poor-risk groups, respectively. (*P* < 0.001). The c-statistic value of our model at 2 years is 0.8 (95% CI, 0.75–0.86).

NLR, mGPS, and PG-SGA were independently related to survival. Our prognostic model using inflammatory- and nutrition-based scores could provide prognostic information to patients and physicians.

## INTRODUCTION

Patients with metastatic gastric cancer (mGC) tend to have miserable prognosis. Although the outcomes of patients with mGC have been shown to improve over time, the median overall survival remains below 1 year.^1^ Currently, a fluoropyrimidine-based plus platinum-based combination chemotherapy with or without a third drug is the standard treatment for patients with mGC. In 2006, a remarkable, randomized, multinational phase III study demonstrated that adding docetaxel to 5-fluorouracil plus cisplatin significantly improved survival and response rate in mGC (23% risk reduction; *P* = 0.02).^2^ For mGC patients with human epidermal growth factor receptor 2 positive, Trastuzumab in combination with chemotherapy exhibits a greater survival benefit than chemotherapy alone (26% risk reduction; *P* = 0.0046).^3^ A novel targeted therapy with Ramucirumab also has survival benefits in mGC patients progressing after first-line chemotherapy (22.4% risk reduction; *P* = 0.047).^4^ Despite the better achievements in biological therapies and medical management, the outcome changes little in the last few decades.

Recent studies have identified the prognostic impact of inflammatory response and nutritional status on survival of patients with gastric cancer. Notably, the neutrophil to lymphocyte ratio (NLR) has been shown to be an independent factor. Graziosi et al^5^ showed elevated preoperative NLR predicts poor overall survival following resection for gastric adenocarcinoma. Mohri et al^6^ verified the high NLR as a predictor for poor prognosis in patients with mGC. Cho et al^7^ also confirmed that pretreatment NLR is a useful prognostic marker in patients with mGC who are undergoing palliative chemotherapy. Interestingly, a latest literature indicated that modified Glasgow prognostic score (mGPS) is a robust predictor of gastric cancer survival as compared with NLR.^8^ mGPS is an inflammation-based score and is calculated on the basis of serum albumin and C-reactive protein (CRP) level. Several prospective cohort studies have also confirmed that mGPS were significant independent predictors of overall survival in patients with advanced cancer.^9,10^ Furthermore, Patient-Generated Subjective Global Assessment (PG-SGA) is a useful tool to access the nutrition status of patients and has been correlated with cancer cachexia and prognosis.^11,12^ Although the prognostic influences of inflammatory response and nutrition status are well-established, no risk model based on these scores has been provided. Therefore, this present study aims to construct a prognostic model to predict survival using inflammation- and nutrition-based scores in patients with metastatic gastric adenocarcinoma treated with chemotherapy.

## PATIENTS AND METHODS

### Patients Selection

Patients who were diagnosed to have mGC from 2007 to 2014 at Kaohsiung Chang Gung Memorial Hospital were reviewed. Inclusion criteria were age >18 years, histologically confirmed gastric adenocarcinoma, integrated information (NLR, mGPS and PG-SGA) within 1 week before chemotherapy, and receiving at least 1 cycle of chemotherapy for their mGC. Chemotherapy regimen was decided at the discretions of physicians. Exclusion criteria were palliative chemotherapy-naïve, incomplete relevant laboratory data, clinical evidence of infection or other inflammation condition, double cancers, and irregular follow-up visiting. After a retrospective chart review, a total 673 patients were pathologically diagnosed to have gastric cancer. Only 281 patients developed metastatic disease in the follow-up period. After excluding those who did not receive palliative chemotherapy, 256 patients were enrolled into our study. This study was approved by the Institutional Review Board of Chang Gung Memorial Hospital.

### Data Collection

Data on patient demographics, Eastern Cooperative Oncology Group performance status (ECOG PS), pathology differentiation, metastatic sites, NLR, mGPS, PG-SGA, first-line chemotherapy regimen for mGC, and survival time after chemotherapy were retrospectively obtained from medical charts. Chemotherapy regimen was decided by physician's discretions. Patients’ inflammatory response and nutritional status were evaluated within 2 weeks before their first cycle of chemotherapy. NLR was calculated as the absolute neutrophil count divided by the absolute lymphocyte count. The cuffoff level of NLR was set to be 3 according to definition of previous literatures.^13,14^ A mGPS score of 2 was assigned if both albumin was <3.5 g/dL and CRP concentration was >10 mg/L, mGPS of 1 if albumin was >3.5 g/dL and CRP concentration was >10 mg/L, and mGPS of 0 if CRP concentration was <10 mg/L despite of albumin level. PG-SGA was also utilized for nutrition stratification by dietitians. This tool was used as described by Read et al.^15^ PG-SGA category A refers to well nourished, category B refers to moderately malnourished, and category C refers to severely malnourished.

### Statistical Analysis

All the clinical variables were presented with frequencies. Continuous variables such as NLR were converted to categorical variables using established cut points. We conducted Cox regression models with “enter” selection to adjust for the effects of potential confounders for univariate and multivariate analyses. It is considered to be statistically significant if *P* < 0.05. A prognostic model was developed by counting the number of these unfavorable features in each patient for predicting prognosis. PG-SGA category A or B was counted as 0, and category C was counted as 1. Similarly, mGPS 0 or 1 was numbered as 0, and mGPS 2 was numbered as 1. NLR <3 was defined as 0 and higher was defined as 1. After computing these scores, patients were classified into favorable-, intermediate-, and poor-risk groups. Kaplan-Meier curves were estimated for overall survival (OS). OS was calculated from the beginnings of chemotherapy until death or the last visiting. Receiver-operating characteristic (ROC) curves and the area under the curve (c-statistic) with 95% confidence interval (CI) were also calculated to access their predicting performances.

## RESULTS

Among these 256 patients with mGC, the median age was 60 years (range: 26–85 years). After a median follow-up visit of 19.4 months (1.9–61.6 months), 77% patients died because of their disease. Table 1 provides a summary of clinical characteristics of our patients. Most patients were male (69%), older than 60 years (55%), better ECOG PS (72%) and well or moderate differentiation (59%). Approximately, 80% patients had liver metastasis, followed by lymph node (72%), peritoneum (31%), lung (14%), bone (11%), and central nerve system (2%). Given the cutoff value of NLR was set to be 3, 130 patients had high NLR (51%) and 126 had low NLR (49%). Meanwhile, around 39% patients were classified into mGPS 1 and 37% into PG-SGA category B. Regarding the treatment of mGC, 85% patients received first-line chemotherapy regimen with fluoropyrimidine- and platinum-based combination, predominantly capecitabine plus oxaliplatin. The remaining 15% patients were treated with fluoropyrimidine-only regimen, such as S-1, capecitabine, uracil-tegafur, and high-dose 5-fluorouracil plus leucovorin.

**TABLE 1:**
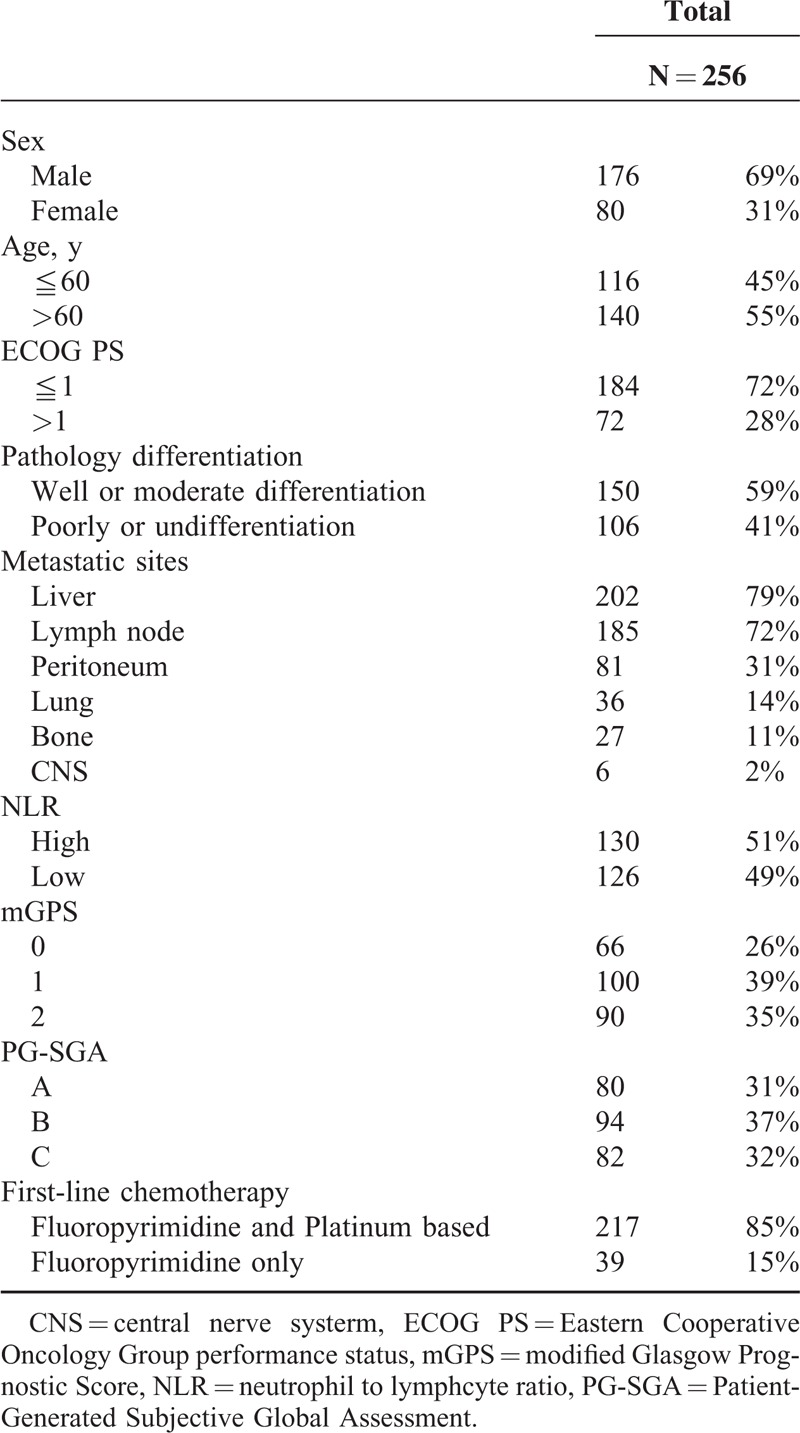
Clinical Characteristics of Patients With Metastatic Gastric Cancer

The Cox regression analyses of OS for all patients are shown in Table 2. Univariate analysis showed that peritoneal metastasis (*P* ≤ 0.001), NLR (*P* ≤ 0.001), mGPS (*P* = <.001), and PG-SGA (*P* = <.001) were significant predictive factors. Furthermore, multivariate analysis also demonstrated that peritoneal metastasis (*P* ≤ 0.006), NLR (*P* ≤ 0.007), mGPS (*P* ≤ 0.001), and PG-SGA (*P* ≤ .001) were positively correlated with OS. The risk model and scoring system of prognostic score generated from β coefficients of multivariate analysis are shown in Table 3. The total prognostic scores range from 0 to 6. A prognostic model to predict survival in patients with mGC treated with chemotherapy was constructed using inflammatory- and nutrition-based scores, including NLR, mGPS, and PG-SGA. Finally, each patient was assigned according to their prognostic scores, including favorable (sum score 0–2), intermediate (sum scores 3–4), and poor (sum scores 5–6) risk groups.

**TABLE 2:**
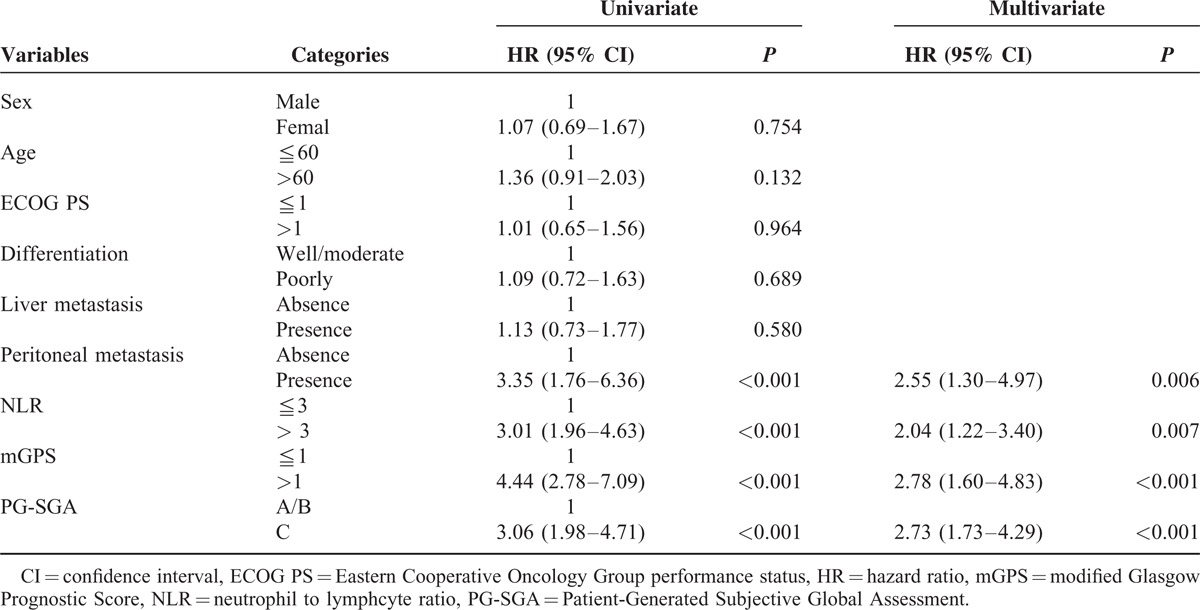
Univariate and Multivariate Cox Regression Analysis of Parameters Associated With Overall Survival

**TABLE 3:**
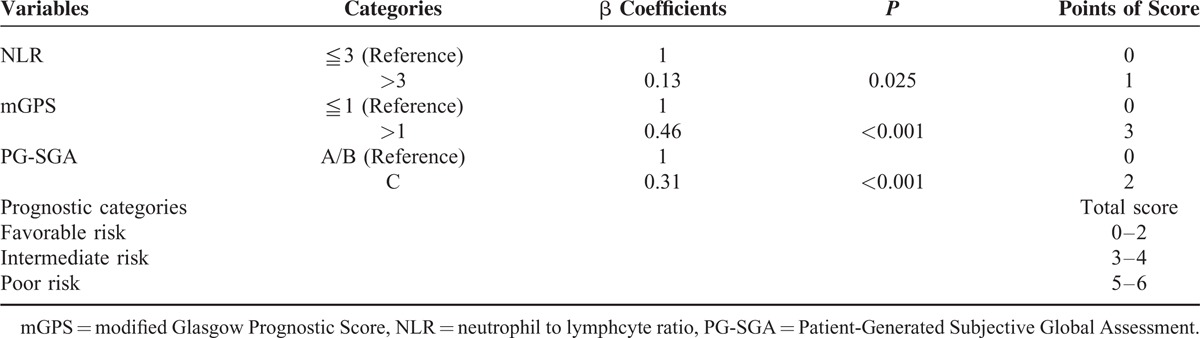
Risk Model and Prognostic Score of Metastatic Gastric Cancer Treated With Chemotherapy

**FIGURE 1:**
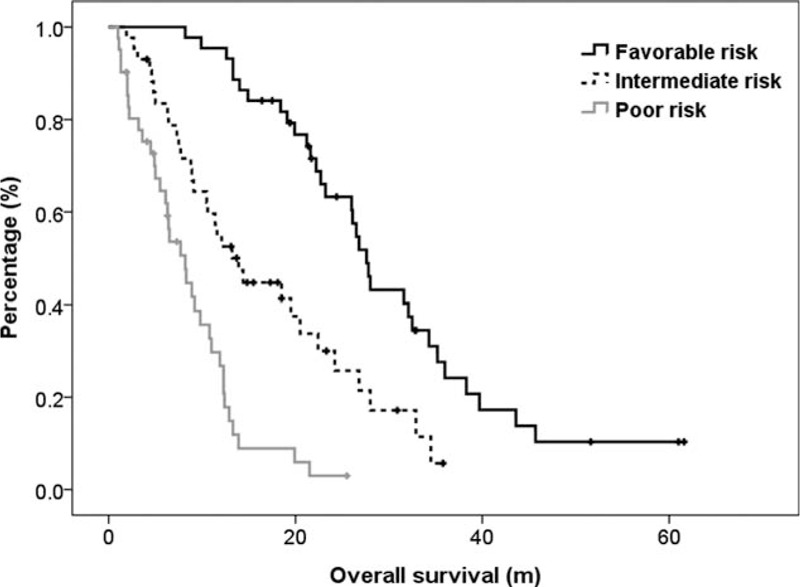
Overall survival of metastatic gastric cancer stratified by risk groups.

Using this risk model, patients were stratified to favorable-risk groups (34%), intermediate-risk group (34%), and poor-risk group (32%). Table 4 provided the oncologic outcomes of mGC treated with chemotherapy stratified by risk groups. The median OS were 27.6 versus 13.2 versus 8.2 months in favorable-, intermediate-, and poor-risk groups, respectively. The 1-year survival rate was 47% versus 27% versus 11%, 2-year survival rate was 26% versus 8% versus 1%, and 3-year survival rate was 9% versus 0% versus 0% in favorable-, intermediate-, and poor-risk groups, respectively. There were strong significant differences in OS and survival rates between these 3 groups (*P* < 0.001). The c-statistic value of this risk model was 0.63. The survival curves were depicted in Figure 1.

**TABLE 4:**
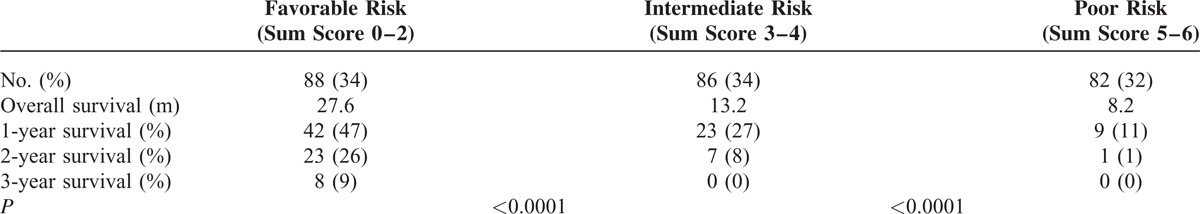
Survival Outcomes of Metastatic Gastric Cancer Stratified by Risk Groups

**FIGURE 2:**
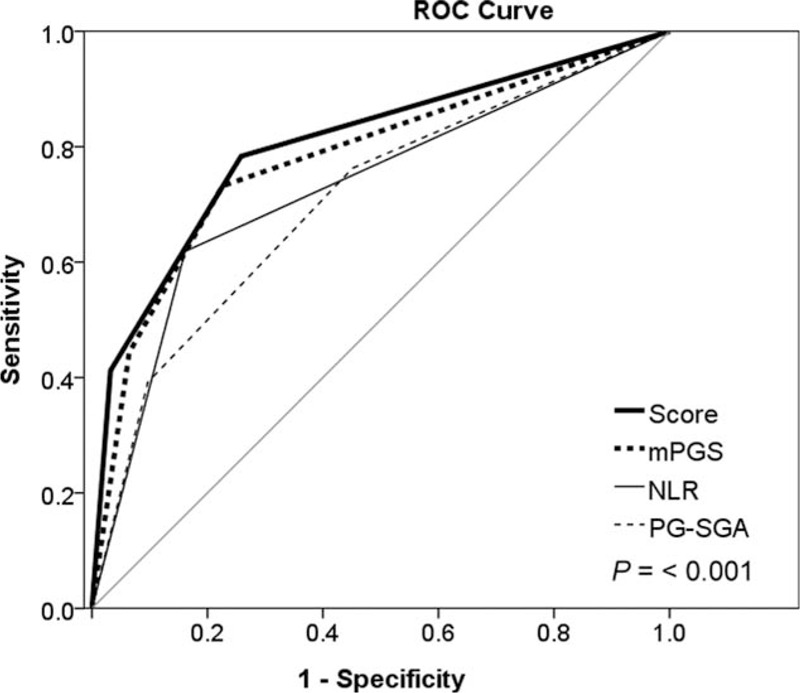
Receiver-operating characteristic curve using the prognostic score and other variables for the outcome of 2-year mortality.

The ROC curve analysis for outcomes at 1, 2, and 3 years using the prognostic score were significantly higher than mGPS, NLR, PG-SGA alone Table 5. The c-statistic at 1 year was 0.84 (95% CI, 0.79–0.88) for the prognostic score compared with 0.83 (95% CI, 0.77–0.88), 0.69 (95% CI, 0.62–0.75), and 0.73 (95% CI, 0.66–0.79) for mGPS, NLR and PG-SGA, respectively (*P* < 0.001). At 2 years, the c-statistic for the prognostic score was 0.80 (95% CI, 0.75–0.86) compared with 0.78 (95% CI, 0.72–0.84), 0.73 (95% CI, 0.66–0.80), and 0.71 (0.64–0.78) for mGPS, NLR, and PG-SGA, respectively (*P* < 0.001). At 3 years, the c-statistic for prognostic score was 0.85 (95% CI, 0.79–0.90) compared with 0.83 (95% CI, 0.76–0.89), 0.77 (95% CI, 0.69–0.85), and 0.78 (0.69–0.87) for mGPS, NLR, and PG-SGA, respectively (*P* < 0.001). Figure 2 plotted the ROC analysis using the prognostic score and other variables for the outcome of 2-year mortality.

**TABLE 5:**
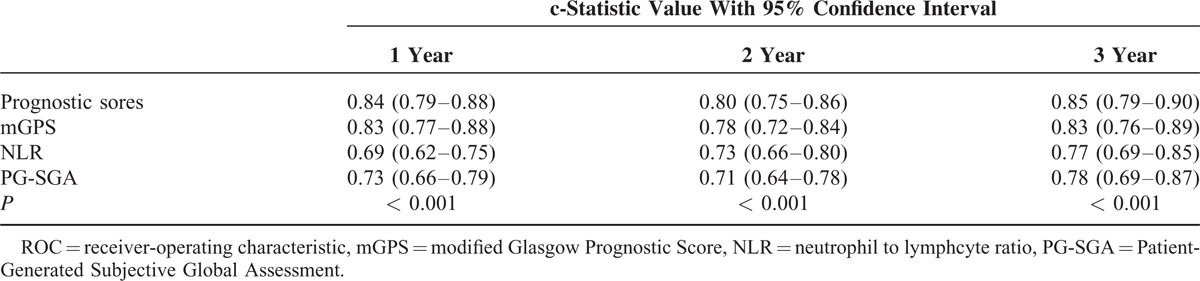
Comparison of c-Statistic Value at 1, 2, 3 Years Using ROC Curve Analysis

## DISCUSSION

The prognosis of patients with mGC is poor. Standard catatonic chemotherapy with fluoropyrimidine- and platinum-based regimen is typically used as first-line treatment for mGC, with median survival no >1 year.^16^ Therefore, it is clinically valuable to identify significant variables to predict survival. Base on this present study, we retrospectively constructed a prognostic model using inflammatory- and nutrition-based scores in patients with mGC treated with chemotherapy, including NLR, mGPS, and PG-SGA. After scoring, each patient was classified into favorable-, intermediate-, and poor-risk group. The median OS and survival rates were all significantly different. Tan et al^9^ also confirms the positive relationship between nutritional status, inflammatory markers, and survival in patients with advanced cancer. We believe this is a reliable prognostic model and is useful for anticipation of outcomes, risks stratification in clinical trials as well as patients counseling. Further external validations are warranted to confirm our conclusion.

Recently, there are growing interests in exploring the relationships between inflammatory response and oncologic outcomes. The NLR in the peripheral blood has been demonstrated to be a prognostic factor in various kinds of cancers,^17–20^ including gastric cancer.^7,21^ The cutoff value of NLR was inconclusive. Jung et al^13^ suggested that cutoff value 3.0 showed a significant prognostic effect on disease-free survival (hazard ratio = 1.654; 95% CI, 1.088–2.515; *P* = 0.019). Shimada et al^14^ also showed NLR >3 was significant for predicting survival. Thus, our study defines NLR 3 according to aforementioned literatures. The possible mechanism for the poor prognosis in patients with high NLR remains under investigation. One explanation is that neutrophil may play both promotion of cancer cell growth and metastasis and/or suppression of lymphocyte activity.^22^ Another explanation is decreased lymphocyte count leads to diminish host's cellular adaptive immunity against cancer cells as well as unable to attack cancer cells and eliminate nascent tumor cells.^23^

Another scoring system of interest is the combination of CRP and albumin, named mGPS. CRP is a highly sensitive, but nonspecific inflammatory marker that can be expressed by several cancer cells. It is widely accepted that an elevated CRP level indicates a more aggressive malignant potential and worsen outcomes. Hypoalbuminemia is known to be secondary to systemic inflammatory response. In particular, previous studies had elucidated that hypoalbuminemia is significantly associated with poor prognosis in patients with gastric cancer.^24,25^ The reason is that elevated CRP levels and hypoalbuminemia have been shown to correlate with upregulation of the inherent immune system, including complement and macrophage function.^25^ Thus, mGPS can reflect both the inflammatory response, nutritional status in patients with mGC, and predicts the survival outcomes.^10^

For decades, nutrition has been an important issue in patients with all kinds of cancer. Several tools were developed to evaluate nutritional status for outcomes anticipation. Body mass index (BMI) is one of the commonly used tool and was estimated by physical condition.^26^ Moreover, Martin et al^27^ suggest Cancer cachexia with skeletal muscle depletion is also a strongly prognostic factor, independent of BMI. However, the Oncology Nutrition Dietetic Practice Group of the American Dietetic Association and Oncology Nursing Society recommend the use of the PG-SGA for evaluation about nutritional status in patients with all kinds of cancer.^28^ The PG-SGA was assessed with a questionnaire about the weight of patients, intake, symptoms, and functional ability, along with a detailed physical examination by physicians and scoring of metabolic abnormalities.^11^ PG-SGA is a convenient nutrition assessment tool that allows for stratification and screening of malnutrition patients with cancer.^29^

Our study is a retrospective chart review and may have several potential biases. Meanwhile, one single-institutional experience, a small size of our cohort, variable of chemotherapy regimens and inconsistent follow-up duration, also limit the power of our study. Furthermore, our prognostic model needs to be validated in a larger patient cohort externally before clinical use. Given these inevitable selection biases, which are inherent to any retrospective studies, our work helps improving survival prediction for patients with mGC treated with chemotherapy by using inflammatory- and nutrition-based scores.

In conclusion, this retrospective study identified several predictive factors for patients with mGC treated with chemotherapy, including NLR, mGPS, and PG-SGA. Based on these variables, a prognostic model was developed using inflammatory- and nutrition-based scores. The OS and 2-year survival rate are significantly different between these risk groups. Taken together, our study is clinically useful for outcomes prediction, consultation as well as risks stratification in clinical trials.
